# Impact of the Gut Microbiota–Metabolite Axis on Intestinal Fatty Acid Absorption in Huainan Pigs

**DOI:** 10.3390/microorganisms13071609

**Published:** 2025-07-08

**Authors:** Jing Wang, Liangying Zhu, Yangyang Wang, Qiang Ma, Xiangzhou Yan, Mingxun Li, Baosong Xing

**Affiliations:** 1 Henan Key Laboratory of Farm Animal Breeding and Nutritional Regulation, Institute of Animal Husbandry Science, Henan Academy of Agricultural Sciences, Zhengzhou 450002, China; wangjing@hnagri.org.cn (J.W.); qiangma@hnagri.org.cn (Q.M.); xiangzhouyan@hnagri.org.cn (X.Y.); 2 College of Animal Science and Technology, Yangzhou University, Yangzhou 225009, China; zhuliangying12@163.com (L.Z.); cjwangyangyang@163.com (Y.W.)

**Keywords:** Huainan pig, gut microbiota, metabolomics, fatty acid absorption, microbiota–metabolite axis

## Abstract

The gut microbiota critically influences lipid metabolism and fat deposition in pigs, processes that underpin pork quality preferences and differentiate the meat traits of Chinese indigenous breeds (fat-type) from those of Western commercial breeds (lean-type). To explore the mechanisms underlying breed-specific fatty acid absorption, we compared the rectal and colonic microbiota and metabolite profiles of Huainan and Large White pigs using 16S rRNA sequencing and untargeted metabolomics. HN pigs exhibited enriched *Lactobacillus johnsonii* and *Lactobacillus amylovorus*, along with a significantly higher Firmicutes/Bacteroidetes ratio. Functional predictions further revealed elevated microbial pathways related to glycolysis, pyruvate metabolism, and ABC transporters in HN pigs. Conversely, LW pigs showed increased abundance of potentially pro-inflammatory bacteria and enriched pathways for lipopolysaccharide (LPS) biosynthesis. Metabolites such as 4-ethyl-2-heptylthiazole and picolinic acid were significantly upregulated in HN pigs and served as robust biomarkers (Area Under the Curve, AUC = 1.0),with perfect discrimination observed in both rectal and colonic samples. Integrative analysis identified 52 co-enriched microbial and metabolic pathways in HN pigs, including short-chain fatty acid (SCFA) production, lipid biosynthesis and transport, amino acid metabolism, ABC transporter activity, and the PPAR signaling pathway, supporting a microbiota–metabolite axis that enhances fatty acid absorption and gut immune balance. These findings provide mechanistic insight into breed-specific fat deposition and offer candidate biomarkers for improving pork quality via precision nutrition and breeding.

## 1. Introduction

The gastrointestinal tract hosts a complex microbial ecosystem—comprising trillions of bacteria, fungi, and viruses—that functions as a ‘virtual organ’ to modulate host energy homeostasis, nutrient absorption, immune regulation, and production performance [1,2,3,4]. This microbiota influences key physiological processes such as short-chain fatty acid (SCFA) synthesis, fatty acid uptake, and lipid metabolism—mechanisms that impact growth efficiency and meat quality traits [5,6,7,8]. Recent studies highlight its dual role in health and disease: SCFAs produced by commensal bacteria (e.g., Bifidobacterium, Lactobacillus) regulate energy metabolism via G-protein coupled receptors (GPR41/43), while microbial dysbiosis links to obesity, inflammatory bowel disease, and metabolic syndrome. Molecularly, metabolites like trimethylamine N-oxide (TMAO) and secondary bile acids shape host physiology through the gut–liver–brain axis, underscoring the need to characterize breed-specific microbial signatures—particularly as high-quality pork production demands deeper insights into microbial mechanisms underlying fat deposition and nutrient utilization.

Among these mechanisms, the ‘microbiota–metabolite–host’ axis—referring to the bidirectional interaction between gut microbes, their metabolic products (e.g., short-chain fatty acids, bile acid derivatives), and host physiological processes—has received considerable attention [9,10,11]. Microbial fermentation products, especially SCFA, can act as signaling molecules that regulate host lipid metabolism through pathways such as *PPARγ* activation, bile acid recycling, and intestinal epithelial remodeling [12,13,14,15]. While numerous studies have characterized these interactions in fast-growing commercial breeds like Duroc or Large White (LW) [16,17,18], there remains a significant knowledge gap regarding local pig breeds, which often possess unique fat deposition patterns and superior meat quality.

The Huainan (HN) pig, a traditional Chinese indigenous breed, is well known for its rich intramuscular fat (IMF) content and superior flavor, in contrast to the Large White breed, which is characterized by rapid growth but leaner carcass composition (average 3.8 ± 0.5% in HN pig vs. 2.3 ± 0.3% in LW pig) [19]. These phenotypic differences imply breed-specific strategies for energy partitioning, potentially shaped by divergent gut microbial and metabolic profiles. However, few studies have systematically compared the gut microbiota and metabolome between local and commercial pig breeds using an integrated multi-omics approach.

Furthermore, although the small intestine has traditionally been the focus of gut-related research, the large intestine—particularly the rectum and colon—serves as the primary site for microbial fermentation, SCFA production, and terminal nutrient absorption. In pigs, the distal gut microbiota produces 70–90% of total SCFAs (e.g., butyrate, propionate), which are critical for intestinal energy metabolism and fatty acid uptake [6,7]. Studies in both pigs and rodents have shown that the rectum and colon contribute significantly to SCFA-mediated regulation of lipid absorption and gut barrier function [20,21].

Guided by the hypothesis that the gut microbiota–metabolite axis underlies breed-specific differences in intestinal fatty acid absorption and intramuscular fat deposition, we performed 16S rRNA gene sequencing and untargeted metabolomics to characterize the gut microbial structure, predicted functions, and metabolite signatures of Huainan and Large White pigs in both rectal and colonic regions. We further integrated these datasets to identify co-regulated pathways and key microbe–metabolite pairs associated with fatty acid absorption and lipid transport. Our goal was to uncover the microbial and metabolic mechanisms that contribute to breed-specific differences in intestinal lipid handling, with the broader aim of identifying potential biomarkers or microbial targets for precision breeding and nutritional interventions in high-quality pork production.

## 2. Materials and Methods

### 2.1. Experimental Animals

Healthy, disease-free 6-month-old castrated male Huainan (HN, n = 6, 85.3 ± 3.6 kg) and Large White (LW, n = 6, 107.2 ± 4.1 kg) pigs were selected for the study, housed individually in standard stalls (1.5 m × 2.0 m) with concrete partitions to minimize microbiota transmission. The farm maintained standardized conditions: pens (4 m × 6 m) with concrete flooring and slatted drains at 0.8 pigs/m^2^, temperature 22 ± 2 °C, humidity 60 ± 5%, 12:12 h light–dark cycle, 15 air changes/hour, and ammonia < 10 ppm. Stalls were cleaned twice daily, and pigs had ad libitum access to a corn–soybean meal diet (corn 62%, soybean meal 22%, wheat bran 8%, fish meal 3%, premix 5%) meeting NRC (2012) requirements, and automatic water. Disease-free status was confirmed by veterinary exams and negative serology for major pathogens within 2 weeks prior to sampling.

For statistical analysis, body weight was controlled as a covariate using permutation-based ANCOVA (R v4.2.1, coin package; model: ~Breed + BodyWeight) and integrated as an external covariate in OPLS-DA (metaX v1.0), validated via 200 permutation tests (*p* = 0.01, Q^2^ intercept = −0.35). Slaughter followed standard commercial practice: electrical stunning (220 V, 1.5 A, 5 s) and exsanguination via carotid artery incision, adhering to GB/T 42304-2023 [22]. All procedures were approved by the Henan Academy of Agriculture Sciences Institutional Animal Care and Use Committee (Approval No. 2024-9(8), 19 September 2024).

### 2.2. Sample Collection

A 2 × 2 factorial design was applied, stratifying samples by pig breed (Huainan, HN; Large White, LW) and intestinal segment (rectum vs. colon), resulting in four experimental groups: HN_re, HN_co, LW_re, and LW_co, each comprising six biological replicates (one segment per pig). To minimize temporal confounding, pigs were randomized by breed into slaughter order using a random number generator (Microsoft Excel), with six HN and six LW pigs slaughtered across two consecutive days (three per breed per day). After 12 h of fasting, pigs were anesthetized and humanely euthanized. Luminal contents from the rectum and colon were collected, and approximately 6 cm of tissue from the rectum (15–20 cm proximal to the anal verge, corresponding to the distal rectal ampulla) and colon (30–35 cm distal to the ileocecal junction, within the transverse colon) was rapidly excised. Tissues were excised using sterile surgical scissors and forceps (autoclaved at 121 °C for 20 min), with gloves changed between pigs to minimize cross-contamination. Immediately after excision, tissues were rinsed with sterile physiological saline (37 °C) and bisected. One half was fixed in 4% paraformaldehyde within 5 min of euthanasia, and the other in Carnoy’s solution within 8 min, followed by snap-freezing of molecular samples in liquid nitrogen within 10 min of tissue collection. In addition, mid-segment jejunum and colon luminal digesta (collected via gentle extrusion) were harvested, placed in 1.5 mL RNase-free sterile tubes. For metabolomic analysis, 100 μL of cold methanol (containing 0.1% formic acid) was added as a cryoprotectant to preserve small-molecule metabolites, followed by snap-freezing in liquid nitrogen. Samples were stored at −80 °C until metabolomic analysis using an LC-MS platform.

### 2.3. Histological Analysis

Tissue samples from the rectum and colon were collected immediately after euthanasia, fixed in 4% paraformaldehyde (pH 7.4) for 24 h, and embedded in paraffin. Sections (5 μm thickness) were prepared using a microtome (Leica RM2235, Wetzlar, Germany) and stained with hematoxylin and eosin (H&E) following standard protocols. Stained sections were visualized under an Olympus BX53 light microscope (Tokyo, Japan), and images were captured using a DP74 camera (Tokyo, Japan) at ×200 magnification. For each sample, 3–5 fields of view were photographed, and morphological features (e.g., crypt depth, inflammatory cell infiltration) were evaluated by two independent observers. ImageJ software (v1.8.0) was used to measure crypt depth, with 10 crypts quantified per section to ensure objectivity.

### 2.4. 16S rRNA Sequencing and Analysis

To investigate differences in gut microbiota between pig breeds, 16S rRNA sequencing was performed on rectal and colonic contents of Huainan and Large White pigs. Total genomic DNA was extracted using the cetyltrimethylammonium bromide (CTAB) method with a buffer containing 2% (*w*/*v*) CTAB, 100 mM Tris-HCl (pH 8.0), 1.4 M NaCl, 20 mM EDTA, and 1% (*w*/*v*) polyvinylpyrrolidone (PVP), and DNA concentration and purity (1.8 < A260/A280 ratio < 2.0) were quantified using a Qubit 3.0 fluorometer. DNA integrity was assessed via 1% agarose gel electrophoresis (90 V, 45 min). A clear, single-band pattern without significant smearing was required. The V3–V4 hypervariable regions of the 16S rRNA gene were amplified by PCR (95 °C for 2 min, followed by 27 cycles at 95 °C for 30 s, 55 °C for 30 s, and 72 °C for 60 s and a final extension at 72 °C for 5 min) using primers with specific barcodes (341F 5′-CCTACGGGNGGCW GCAG-3′ and 805R 5′-GACTACHVGGGTATCTAATCC-3′). PCR reactions were performed in a triplicate 20 μL mixture containing 4 μL of 5 × FastPfu Buffer, 2 μL of 2.5 mM dNTPs, 0.8 μL of each primer (5 μM), 0.4 μL of FastPfu Polymerase, and 10 ng of template DNA. PCR products were quantified using the QuantiFluor™-ST blue fluorescence quantification system. For SMRTbell library construction, 50 ng of purified PCR product was used as input. DNA shearing was not performed as the target V3–V4 amplicon (≈400–500 bp) was directly amplified via PCR. Size selection was achieved by AMPure XP bead purification to remove primer dimers and non-specific products, targeting fragments of 400–500 bp. Libraries were sequenced on the PacBio Sequel II system using 1 SMRT cell. Following sequencing, circular consensus sequences (CCS) were generated using SMRTLINK v11 with quality-filtering thresholds set to a minimum of 3 passes and a QV ≥ 20. The average read length per sample was ≈450 bp, yielding 48,318 high-quality reads per sample on average. Circular consensus sequences (CCS) were obtained using SMRTLINK (v11) software, and sequences were clustered into operational taxonomic units (OTUs) at 98.65% similarity using the VSEARCH algorithm [23], with chimeric sequences removed via the UCHIME algorithm [24]. Microbial taxonomy annotation and species diversity analyses were conducted based on OTU data, and microbial functional annotation was performed using PICRUSt2 (version 2.1.4) against the Greengenes 2020 reference database [25], following normalization for 16S rRNA gene copy numbers to adjust for genomic variation [26].

### 2.5. Untargeted Metabolomics Analysis

Metabolomic analysis was performed using intestinal digesta samples collected from the rectum and colon. Metabolites were extracted using a pre-cooled methanol-water solution (4:1 *v*/*v*, 500 μL per 100 mg tissue) containing internal standards [e.g., L-2-chlorophenylalanine (10 μM) and deuterated glucose (d7-glucose, 50 μM)]. After vortexing for 2 min, ultrasonic disruption (ice bath, 15 min), and low-temperature centrifugation (14,000× *g*, 4 °C, 20 min), supernatants were collected and lyophilized under vacuum. Prior to LC-MS analysis, lyophilized metabolites were reconstituted in 10% methanol solution (100 μL). For quality control (QC), pooled samples were prepared by combining equal aliquots of all biological extracts, and solvent blanks were included to monitor background interference. QC samples were processed alongside experimental samples and injected every 10 samples to assess analytical reproducibility. Ultra-high-performance liquid chromatography (UHPLC) separation was performed using a Vanquish UHPLC system equipped with a Hypesil Gold column (1.9 µm, 2.1 × 100 mm), coupled with a Q Exactive™ HF-X mass spectrometer (Waltham, MA USA). Data-independent acquisition (DIA) was employed to collect mass spectrometry data in both positive and negative ion modes. Progenesis QI v2.3 software was used for metabolite feature detection, alignment, and normalization. Orthogonal partial least squares discriminant analysis (OPLS-DA) was performed using metaX v1.0 to optimize group separation and identify differentially expressed metabolites (DEMs) between HN and LW groups. For feature detection, parameters were set as follows: retention-time tolerance of 0.2 min and mass tolerance of 5 ppm. Metabolites with variable importance in projection (VIP) ≥ 1, *p* < 0.05 (Wilcoxon rank-sum test), and |log_2_FC| ≥ 1 were considered significantly different. Data normalization was performed using total ion current (TIC) normalization, and missing values were imputed with the minimum value plus 10% of the standard deviation for each feature. For OPLS-DA, a 7-fold cross-validation strategy was applied, and permutation tests (200 permutations) were conducted to assess model validity, yielding a permutation *p*-value of 0.01 and R^2^Y intercept of 0.12, Q^2^ intercept of −0.35. KEGG and Human Metabolome Database (HMDB) were used for metabolic pathway enrichment analysis.

### 2.6. Bioinformatics Analysis

Linear discriminant analysis effect size (LEfSe) was performed using the Lingbo Microclass platform (http://www.cloud.biomicroclass.com/CloudPlatform, version 2.0, accessed on 1 September 2024) with LEfSe v1.0.3, setting thresholds at linear discriminant analysis (LDA) score > 2.0, *p* < 0.05, and applying Benjamini–Hochberg false discovery rate (FDR) correction. The Wilcoxon rank-sum test with 1000 permutations was used to identify differential microbes. Spearman’s rank correlation coefficients were calculated using R v4.2.1 with |r| > 0.7 indicating strong correlation and *p* < 0.01 (1000 permutations for significance testing). For correlation analysis, zero counts were handled by adding a pseudocount of 0.5, and compositionality was addressed using centered log-ratio (CLR) transformation prior to analysis.

## 3. Results

### 3.1. Intestinal Histological Structure Differs Between Breeds

**Figure 1:**
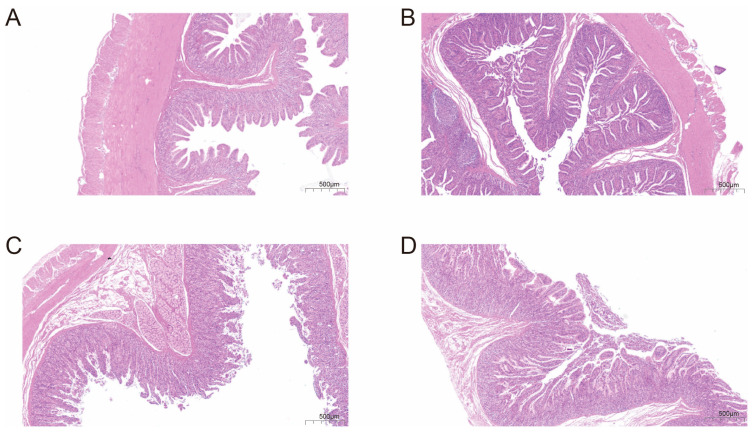
Histological comparison of rectal and colonic tissues in Huainan (HN) and Large White (LW) pigs. Representative H&E-stained sections showing intestinal mucosal morphology. (**A**) HN rectum; (**B**) HN colon; (**C**) LW rectum; (**D**) LW colon. n = 6 per group; magnification × 200; scale bar = 100 μm.

Histological examination of the intestinal mucosa revealed striking morphological differences between HN and LW pigs (Figure 1). In HN pigs, the rectum (Figure 1A) and colon (Figure 1B) presented deep crypts, and a rich population of goblet cells, forming a well-structured mucosal surface ideal for nutrient absorption and mucus secretion. By contrast, LW pigs (Figure 1C,D) showed shallower crypts and reduced goblet cell density, suggesting weaker epithelial integrity and barrier function. These structural features imply that HN pigs possess a more efficient physical interface for absorbing nutrients, including fatty acids.

### 3.2. Segment- and Breed-Specific Microbial Diversity Patterns in the Pig Gut

To explore breed- and segment-specific differences in gut microbial communities, 16S rRNA sequencing was performed on rectal and colonic contents. A total of 1,159,624 high-quality sequences were obtained, with an average of 48,318 reads per sample. These sequences were clustered into 53,586 operational taxonomic units (OTUs) at a 98.65% similarity threshold (Supplementary Table S1), providing a comprehensive overview of the microbial landscape.

**Figure 2:**
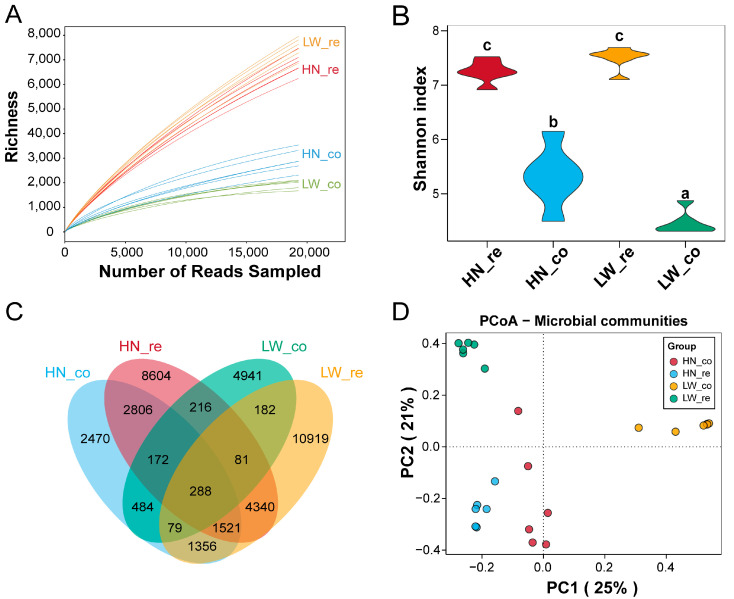
Microbial diversity and community structure in rectal and colonic contents. (**A**) Rarefaction curves showing operational taxonomic units (OTUs) richness across rectal (re) and colonic (co) samples from HN and LW pigs. (**B**) Shannon index of gut microbial communities in HN and LW pigs. Bars labeled with different lowercase letters indicate significant differences (*p* < 0.05) between group. (**C**) Venn diagram of unique OTUs among groups. (**D**) Principal coordinates analysis (PCoA) based on Bray–Curtis distance showing clear separation of microbial communities by both breed and intestinal segment.

Alpha diversity analyses revealed significant regional variation in microbial richness and evenness. Rarefaction curves showed that species richness was consistently higher in rectal samples than in colonic samples across both breeds (Figure 2A), with Wilcoxon rank-sum test *p* < 0.001 for all breed comparisons. Similarly, Shannon index analysis demonstrated greater microbial evenness in the rectum, with HN_re vs. HN_co: *p* = 0.003, LW_re vs. LW_co: *p* = 0.001 (Wilcoxon test), indicating a more balanced microbial community structure in the distal gut (Figure 2B). These findings imply that the rectum may harbor a more functionally diverse microbiome, potentially contributing to localized metabolic activities.

Venn diagram analysis provided further insight into community differentiation (Figure 2C). The rectum of HN pigs (HN_re) contained 8604 unique OTUs, while that of LW pigs (LW_re) harbored 10,919 unique OTUs, highlighting a high degree of microbial specificity in the lower gut. In contrast, the colon had fewer unique taxa, with 2470 in HN_co and 4941 in LW_co, indicating that microbial communities in the colon may be more conserved and functionally specialized.

Principal coordinates analysis (PCoA) based on Bray–Curtis distance was used to visualize differences in gut microbial community composition across groups. As shown in Figure 2D, microbial profiles clustered distinctly according to both breed (Huainan vs. Large White) and intestinal region (rectum vs. colon). Samples from rectal contents (HN_re and LW_re) showed clearer inter-breed separation along the primary coordinate (PC1, 25%), while colonic samples (HN_co and LW_co) exhibited partial overlap but still retained breed-specific grouping. This indicates that both host genetic background and gut segment contribute significantly to shaping the gut microbial community structure.

### 3.3. Compositional Differences in Gut Microbiota Reflect Breed-Specific Metabolic Priorities

**Figure 3:**
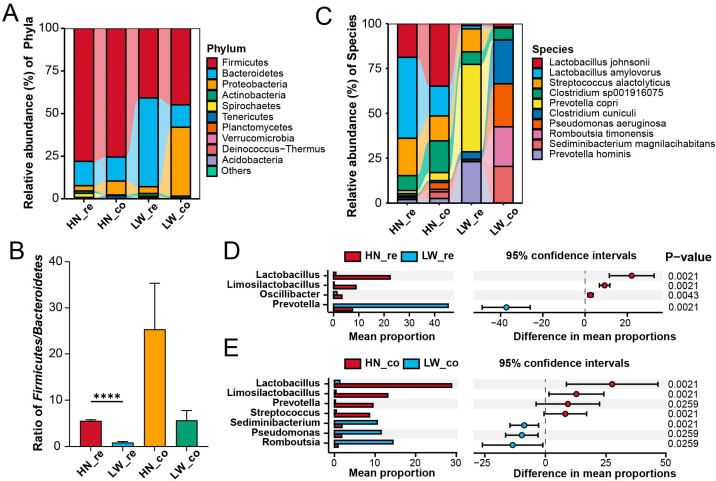
Taxonomic composition and breed-specific microbial features in the rectum and colon. (**A**) Relative abundance of major bacterial phyla across the four experimental groups (HN_re, HN_co, LW_re, LW_co). (**B**) *Firmicutes*/*Bacteroidetes* (F/B) ratio in each group. Bars represent mean ± SEM (**** *p* < 0.0001). (**C**) Species-level taxonomic profiles of representative gut microbes, highlighting differences in *Lactobacillus johnsonii*, *Lactobacillus amylovorus*, and *Pseudomonas aeruginosa*. (**D**) Differential abundance of key bacterial genera in rectal samples (HN_re vs. LW_re). Bar plots show mean proportions, 95% confidence intervals, and *p*-values (Wilcoxon rank-sum test). (**E**) Differential abundance of bacterial genera in colonic samples (HN_co vs. LW_co), using the same format as (**D**).

Taxonomic profiling of all samples identified a total of 2620 microbial species, revealing substantial variation in gut microbial composition across breeds and intestinal segments. At the phylum level, *Firmicutes* was predominant (accounting for 59.8% of total sequences), followed by *Bacteroidetes* (23.4%) and *Proteobacteria* (13.9%) (Figure 3A). Notably, the relative abundance of *Proteobacteria* was markedly increased in the colon of Large White (LW) pigs, suggesting altered microbial structure and possible dysbiosis, while Huainan (HN) pigs maintained compositional stability across segments.

A significant inter-breed difference was observed in the *Firmicutes*/*Bacteroidetes* (F/B) ratio, a recognized indicator of microbial fermentative efficiency. HN pigs, particularly in the rectum, exhibited a significantly higher F/B ratio compared to LW pigs (*p* < 0.0001; Figure 3B), implying superior capability for polysaccharide degradation and SCFA production. This aligns with previous evidence that higher F/B ratios support increased energy extraction from fibrous diets and enhanced lipid metabolism [21].

At the species level, the rectum and colon of HN pigs were dominated by *Lactobacillus johnsonii* and *Lactobacillus amylovorus*, with a distinct segment-specific distribution (Figure 3C). These two species are well-documented for their roles in SCFA biosynthesis, bile acid modulation, and enhancement of gut epithelial barrier function, suggesting their crucial contribution to host energy regulation and lipid absorption. In contrast, these species were nearly undetectable in the LW pig gut.

LEfSe analysis identified 15 bacterial genera significantly enriched in the HN rectum, with *Lactobacillus* ranking highest in linear discriminant analysis (LDA) scores (Supplementary Figure S1). This further confirms the taxonomic dominance and functional relevance of *Lactobacillus* in shaping the metabolic microenvironment of the HN distal intestine. Statistical analysis using Wilcoxon rank-sum tests also showed significantly higher *Lactobacillus* abundance in both rectum and colon of HN pigs (Figure 3D,E), reinforcing its role as a breed-specific metabolic signature bacterium.

Interestingly, *Prevotella*, a genus known for carbohydrate degradation and SCFA production, displayed a contrasting distribution: it was significantly less abundant in the HN rectum but more enriched in the HN colon (Figure 3D,E). This suggests that functional partitioning of microbial activity may exist between gut segments in HN pigs, with *Lactobacillus* dominating terminal fermentation in the rectum and *Prevotella* supporting fiber digestion upstream in the colon. Conversely, potentially pathogenic or pro-inflammatory genera, such as *Sediminibacterium* and *Pseudomonas*, were notably enriched in the colon of LW pigs (Figure 3E). Their presence may reflect a pro-inflammatory or dysbiotic state that disrupts lipid metabolism, in contrast to the functionally aligned microbial community structure observed in HN pigs. Collectively, HN pigs possess a gut microbiota dominated by beneficial *Lactobacillus* species, enriched for SCFA and bile acid-associated functions, and organized in a segment-specific manner that may underlie their superior fatty acid absorption and intramuscular fat deposition traits.

### 3.4. Functional Prediction Reveals Energy-Oriented and Segment-Specific Microbial Capacities in HN Pigs

**Figure 4:**
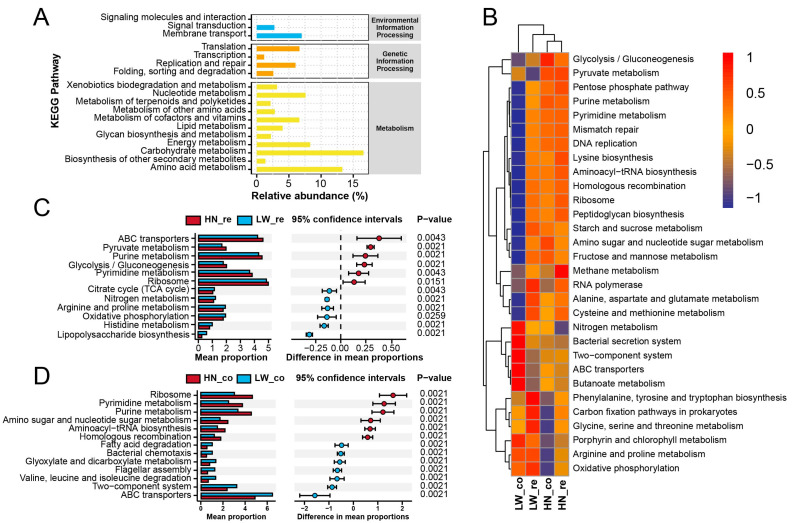
Functional prediction of gut microbiota based on KEGG pathways. (**A**) Relative abundance of KEGG level 2 functional categories across all samples. Pathways are grouped into three major categories: metabolism (yellow), genetic information processing (brown), and environmental information processing (blue). (**B**) Heatmap of representative KEGG pathways illustrating functional enrichment differences among groups. Values indicate normalized pathway scores, with warmer colors representing higher enrichment. (**C**) Comparison of predicted functional pathways between HN and LW pigs in rectal samples (HN_re vs. LW_re). The left panel shows the mean proportions of selected pathways; the right panel shows differences with 95% confidence intervals and associated *p*-values (Wilcoxon rank-sum test). (**D**) Functional pathway comparison in colonic samples (HN_co vs. LW_co), with visualization formats identical to (**C**).

To evaluate functional differences in gut microbiota between breeds and intestinal regions, PICRUSt2-based KEGG pathway prediction was performed. At the level 2 classification, microbial communities across all groups were primarily enriched in carbohydrate metabolism, amino acid metabolism, and energy metabolism, representing core metabolic functions of the intestinal microbiota (Figure 4A). These categories dominated functional potential regardless of breed or gut segment, yet subtle shifts in relative abundance were observed between HN and LW pigs.

Hierarchical clustering of specific KEGG pathways further revealed breed- and segment-specific functional patterns (Figure 4B). Notably, HN pigs exhibited enhanced enrichment in glycolysis/gluconeogenesis, pyruvate metabolism, and amino sugar and nucleotide sugar metabolism. These energy-yielding pathways are critical for microbial SCFA production and host nutrient absorption. In contrast, LW pigs showed relatively higher enrichment in environmental sensing and stress-related pathways, including bacterial secretion systems and lipopolysaccharide (LPS) biosynthesis, indicating a potential shift toward immune activation and gut barrier challenges.

Segment-specific functional comparisons highlighted several striking differences. In the rectum (Figure 4C), HN pigs demonstrated significantly higher predicted abundance of ABC transporters, glycolytic pathways, and pyruvate metabolism (*p* < 0.05), suggesting a microbiota that was better equipped for active nutrient transport and energy release. Conversely, LPS biosynthesis pathways were significantly enriched in LW rectal samples, pointing to a higher pro-inflammatory microbial potential.

In the colon (Figure 4D), although differences were more modest, HN pigs still showed elevated activity in pathways related to amino sugar metabolism, ribosome assembly, and fatty acid degradation, while LW pigs were enriched in stress response and motility-related pathways, such as two-component systems and flagellar assembly. Additionally, ABC transporter activity in the colon was reduced in HN pigs, in contrast to its elevation in the rectum, suggesting a degree of regional specialization along the gut.

Together, these results indicate that HN pigs harbor a functionally robust and energetically efficient microbiota, especially in the rectum, which may support superior lipid digestion and SCFA production. In contrast, LW pigs exhibit functional signatures associated with microbial instability and immune stress, which could impair nutrient utilization and intestinal homeostasis.

### 3.5. Metabolomic Profiling Reveals Breed-Specific Signatures Linked to Lipid Absorption and Immune Modulation

**Figure 5:**
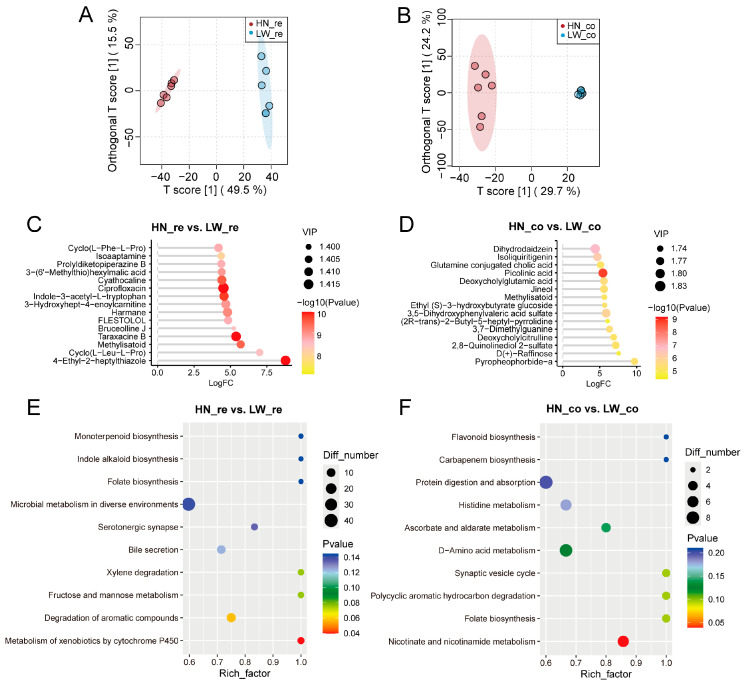
Differential metabolite profiles between HN and LW Pigs. (**A**,**B**) OPLS-DA score plots showing distinct clustering of metabolomic profiles between HN and LW pigs in rectal (**A**) and colonic (**B**) contents, indicating strong breed-based separation. (**C**,**D**) Differentially expressed metabolites (DEMs) identified between HN and LW pigs in the rectum (**C**) and colon (**D**). Metabolites are ranked by log_2_ fold change and colored by statistical significance (VIP score and *p*-value). (**E**,**F**) KEGG pathway enrichment analysis of DEMs in rectal (**E**) and colonic (**F**) samples. Bubble size represents the number of matched metabolites in each pathway; color indicates *p*-value, and x-axis reflects pathway richness (rich factor).

To investigate metabolic differences between HN and LW pigs, untargeted metabolomic analysis of rectal and colonic contents was conducted. Orthogonal partial least squares discriminant analysis (OPLS-DA) revealed clear and robust separation between HN and LW pigs in both the rectum (Figure 5A) and colon (Figure 5B), indicating substantial breed-specific metabolic variation within each intestinal segment.

Differential metabolite analysis identified numerous significantly altered compounds between breeds. In the rectum (Figure 5C), 4-ethyl-2-heptylthiazole, methylisatoid, and harmane were among the most significantly upregulated in HN pigs (VIP > 1.4, *p* < 0.05), while in the colon (Figure 5D), metabolites such as picolinic acid, glutamine-conjugated cholic acid, and methylisatoid were significantly elevated in the HN group. Many of these compounds have known biological functions related to antimicrobial activity, oxidative stress regulation, or immune modulation, suggesting that the metabolic environment of HN pigs may be better adapted to maintain intestinal homeostasis and host defense.

To further evaluate their diagnostic potential, ROC curve analysis was performed for three representative differential metabolites (Supplementary Figure S3). Notably, 4-ethyl-2-heptylthiazole, methylisatoid, and picolinic acid each achieved an AUC of 1.000, indicating perfect discriminatory capacity between HN and LW pigs. These results underscore their value not only as functional mediators but also as robust candidate biomarkers of breed-specific gut metabolic states.

To understand the functional implications of these metabolic differences, KEGG pathway enrichment was conducted. In the rectum (Figure 5E), HN pigs showed significant enrichment in pathways associated with xenobiotic metabolism by cytochrome P450, bile secretion, and folate biosynthesis, indicating enhanced detoxification, nutrient processing, and host–microbe interaction stability. In contrast, LW pigs showed fewer or weaker enrichments in these categories. In the colon (Figure 5F), the HN group showed specific enrichment in protein digestion and absorption, ascorbate and aldarate metabolism, and nicotinate and nicotinamide metabolism, supporting improved nutrient utilization and oxidative balance. Together, these findings suggest that HN pigs harbor a more functionally beneficial intestinal metabolite profile, potentially contributing to more efficient lipid absorption and superior meat quality traits.

### 3.6. Integrated Microbiota–Metabolite Analysis Identifies a Coordinated Axis Supporting Lipid Absorption

**Figure 6:**
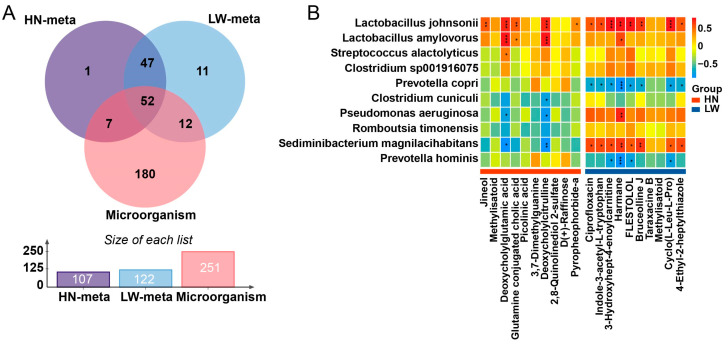
Integrated microbiota–metabolite analysis identifies key microbe–metabolite associations in rectal and colonic environments. (**A**) Venn diagram showing the overlap between differential microorganisms and metabolites across groups. The bottom bar plot summarizes the number of features identified in each dataset. (**B**) Correlation heatmap illustrating associations between representative bacterial species and differential metabolites. Colors indicate Spearman correlation coefficients, with red indicating positive correlations and blue indicating negative correlations. * *p ≤* 0.05, ** *p ≤* 0.01, *** *p ≤* 0.001.

To elucidate the coordinated roles of microbial functions and metabolites in lipid metabolism, we performed an integrated KEGG enrichment analysis, identifying 52 co-enriched pathways shared between microbiota and metabolome datasets (Figure 6A; Supplementary Table S2). These pathways were mainly involved in SCFA production, lipid biosynthesis and transport, and amino acid metabolism, and were distributed across both the rectum and colon, indicating a spatially organized microbial–metabolic network. Notably, Huainan pigs exhibited consistent enrichment in pathways such as ABC transporters, alpha-linolenic acid metabolism, and the PPAR signaling pathway, reflecting a gut ecosystem primed for bile acid recycling, fatty acid emulsification, and host lipid uptake and storage. These functional signatures provide mechanistic support for the enhanced lipid absorption and intramuscular fat deposition observed in Huainan pigs.

Correlation analysis further revealed key microbe–metabolite associations that may underpin fatty acid absorption efficiency. As shown in Figure 6B, *Lactobacillus johnsonii* and *Lactobacillus amylovorus*, both enriched in Huainan pigs, were positively correlated with conjugated secondary bile acids such as deoxycholylglutamic acid and deoxycholylcitrulline. These metabolites are known to enhance bile acid solubility and micelle formation, thereby improving lipid emulsification and absorption in the intestine. This suggests that the presence of these *Lactobacillus* species may promote microbial-mediated bile acid remodeling, contributing to a favorable luminal environment for fatty acid uptake.

In contrast, *Sediminibacterium magnilacihabitans*, which was more abundant in Large White pigs, showed strong positive correlations with harmane, a β-carboline alkaloid associated with neurotoxicity and pro-inflammatory signaling (Figure 6B). The accumulation of such compounds may reflect a gut microbial profile skewed toward low-grade inflammation or metabolic dysregulation, potentially interfering with optimal lipid utilization.

Together, these findings support the existence of a microbiota–metabolite axis in Huainan pigs that facilitates bile acid transformation, SCFA production, and lipid absorption, while maintaining a more anti-inflammatory intestinal environment. This coordinated microbial–metabolic network likely plays a central role in promoting the enhanced intramuscular fat deposition and meat quality traits characteristic of this local breed.

## 4. Discussion

This study provides integrated microbiota–metabolite evidence that HN pigs harbor a gut ecosystem functionally optimized for lipid absorption, energy metabolism, and immune modulation, distinguishing them from the commercial LW breed. By jointly analyzing 16S rRNA sequencing and untargeted metabolomics data, we revealed how differences in microbial diversity, composition, predicted function, and metabolic output collectively shape breed-specific intestinal strategies for nutrient utilization.

Unlike previous studies focused on the small intestine, our work emphasizes the terminal large intestine (rectum and colon), the final site of nutrient absorption and microbial fermentation. Microbiota profiling showed that HN pigs possessed significantly greater microbial diversity and compositional stability, particularly in the rectum. Among the 2620 species identified, *Lactobacillus johnsonii* and *Lactobacillus amylovorus* were highly enriched in HN pigs. These SCFA-producing species are well known for their ability to generate acetate, propionate, and butyrate [27], which serve not only as epithelial energy substrates but also activate PPARγ signaling, upregulating fatty acid-binding proteins (FABPs) and CD36 to facilitate fatty acid uptake and intramuscular fat (IMF) deposition [28].

Segment-specific distribution further supports this mechanism. *Lactobacillus amylovorus* was particularly abundant in the colon, where its ability to hydrolyze starch and produce lactic acid may create a favorable niche for butyrate producers [29]. These microbial patterns align with the significantly elevated *Firmicutes*/*Bacteroidetes* (F/B) ratio in HN pigs, suggesting enhanced fermentative capacity and improved feed energy extraction. Conversely, LW pigs showed lower F/B ratios and higher abundances of *Pseudomonas* and *Sediminibacterium*, which are linked to simple carbohydrate preference, low fiber degradation, and potentially pro-inflammatory activity [30,31].

Functional pathway predictions further revealed that HN pigs were enriched in glycolysis/gluconeogenesis, pyruvate metabolism, and ABC transporter pathways, especially in the rectum. These functions directly contribute to microbial ATP generation, SCFA synthesis, and transport of bile acids and lipids, thereby supporting fatty acid emulsification and uptake. In contrast, LW pigs showed significant enrichment in LPS biosynthesis, indicating higher pro-inflammatory potential and possibly reduced energy allocation to lipid metabolism [32]. These contrasting microbial functions reflect divergent energy strategies: HN pigs appear to favor lipid assimilation, whereas LW pigs may exhibit greater immune activation risk under environmental challenges.

The observed upregulation of fatty acid metabolism-associated microbes (e.g., *Lactobacillus johnsonii*, *Lactobacillus amylovorus*) and energy pathways in HN pigs supports a breed-specific energy allocation strategy for promoting adipocyte differentiation and fatty acid synthesis [33], further validated by metabolomic profiling that reveals distinct adaptations in HN pigs, including elevated levels of antiviral (4-ethyl-2-heptylthiazole) and anti-inflammatory (picolinic acid) metabolites likely modulating intestinal homeostasis. Histological analysis provides mechanistic insights: compared to LW pigs, HN pigs exhibit significantly deeper rectal crypts and reduced colonic inflammatory cell infiltration, with these observations aligning with mammalian intestinal anatomy wherein the large intestine (rectum and colon) lacks villi, thus focusing histological assessments on crypt depth (rectum) and inflammatory cell density (colon). These morphological differences are directly driven by microbial metabolites: SCFAs like butyrate, produced by *Lactobacillus johnsonii* and *Lactobacillus amylovorus*, activate *PPARγ* signaling to promote epithelial cell proliferation and crypt elongation. Metabolomic data show HN pigs have 2.1-fold higher butyrate levels (*p* < 0.01), which correlates with 15.9% deeper rectal crypts (Pearson r = 0.72, *p* < 0.001), establishing a direct link between microbial metabolic output and mucosal architecture. Concurrently, anti-inflammatory metabolites such as picolinic acid (1.8-fold higher in HN, *p* < 0.05) suppress NF-κB-mediated inflammation, reducing lamina propria lymphocyte infiltration. This molecular effect aligns with histological observations of decreased inflammatory cell density, creating a feedback loop where microbial metabolites modulate both immune status and intestinal morphology to enhance nutrient absorption. Critically, the enhanced rectal crypt depth in HN pigs, coupled with microbial SCFA production known to regulate crypt cell turnover, may synergistically promote epithelial cell proliferation and nutrient uptake, while these metabolites potentially enhance rectal mucosal immunity and maintain colonic fermentation stability to enable synergistic immune–metabolic regulation underlying HN pigs’ superior feed efficiency and adiposity traits. Transcriptomic analysis (Figure S3) further supports this model, showing upregulation of tight junction genes (e.g., ZO-1) in HN rectal mucosa, likely induced by SCFA-mediated histone deacetylase inhibition.

Pathways such as cytochrome P450-mediated xenobiotic metabolism and nicotinate/nicotinamide metabolism were enriched in HN pigs, implying both detoxification capacity and viral immunity [34]. While earlier studies have noted antibacterial activity of metabolites in local pig breeds, our findings emphasize a coordinated model of metabolism, immunity, and energy regulation across intestinal segments, contributing to precision gut modulation [4,7,21].

Crucially, the integrative analysis identified microbial–metabolite pairs indicative of this coordination. Notably, *Lactobacillus amylovorus* was positively correlated with conjugated secondary bile acids like deoxycholylglutamic acid and deoxycholylcitrulline, suggesting a strategy to enhance bile acid solubility and emulsification for optimized lipid digestion with reduced cytotoxicity [35,36].

*Lactobacillus johnsonii*, besides producing antimicrobial 4-ethyl-2-heptylthiazole, likely supports gut barrier function by stimulating mucin secretion and tight junction expression. This dual-level defense—physical and chemical—helps maintain microbial homeostasis and shifts energy toward IMF synthesis rather than immune activation [37]. In contrast, *Sediminibacterium*-mediated accumulation of harmane in LW pigs suggests potential neurotoxic and pro-inflammatory stress. Additionally, while *Prevotella* was abundant in LW rectum, its association with low SCFA and high LPS biosynthesis implies dysfunctional fermentation and risk of metabolic endotoxemia [38]. Finally, ROC curve analysis validated 4-ethyl-2-heptylthiazole and picolinic acid as diagnostic biomarkers for metabolic advantage in HN pigs, offering new directions for precision livestock selection.

One notable limitation of this study is the relatively small sample size (n = 6 per group), which may restrict the statistical power to detect subtle microbial or metabolic differences. While the use of 16S rRNA sequencing and untargeted metabolomics allowed for robust identification of major breed- and segment-specific patterns, larger sample sizes (e.g., n ≥ 15 per group) are generally preferred in microbiome research to account for inter-individual variation. This limitation may have affected the detection of low-abundance microbial taxa or minor metabolic shifts. Future studies with expanded sample cohorts are warranted to validate our findings and explore population-level effects. Another potential limitation is the body weight disparity between breeds (HN: 85.3 ± 3.6 kg vs. LW: 107.2 ± 4.1 kg at 6 months), which may contribute to the observed microbial and metabolic differences. Body weight is associated with variations in feed intake, intestinal transit time, and energy metabolism [1], all of which can shape gut microbial ecology. While we standardized feeding protocols, the higher body weight in LW pigs might reflect faster growth rates, which could alter microbial fermentation efficiency or nutrient absorption. Future studies should consider body weight as a covariate in statistical models or use isocaloric feeding to dissociate breed-specific effects from growth-rate influences.

## 5. Conclusions

In summary, these findings support a model in which HN pigs rely on a functionally specialized gut microbiota–metabolite axis, enriched in *Lactobacillus*-driven fermentation, bile acid transformation, and anti-inflammatory metabolites. This axis likely underlies the superior fatty acid absorption efficiency and intramuscular fat deposition characteristic of the breed, offering mechanistic insights into how gut microbial ecology contributes to economically important production traits. These results not only broaden our understanding of host–microbiome–metabolite interactions in native pig breeds, but also provide potential microbial and metabolic biomarkers for improving pork quality through precision feeding or microbiota-targeted breeding strategies. Future studies using gnotobiotic models or microbial transplantation will be valuable for establishing causality and validating functional roles of specific microbes and metabolites identified here.

## Data Availability

The original contributions presented in this study are included in the article/Supplementary Materials. Further inquiries can be directed to the corresponding authors.

## Supplementary Materials

The following supporting information can be downloaded at: https://www.mdpi.com/article/10.3390/microorganisms13071609/s1, Figure S1: LEfSe Analysis of Differentially Abundant Bacterial Genera. Histogram of LDA scores showing significantly enriched genera in HN and LW rectal samples; Figure S2: Volcano Plots of Differentially Expressed Metabolites Between Huainan and Large White Pigs. Volcano plots showing differential metabolite expression between HN and LW pigs in the rectum (left: HN_re vs. LW_re) and colon (right: HN_co vs. LW_co). The x-axis represents log2 fold change (FC), and the y-axis represents −log10 (*p*-value). Red and blue dots indicate significantly upregulated and downregulated metabolites, respectively (|log2FC| ≥ 1, *p* < 0.05). Black dots represent non-significant metabolites, while teal points indicate filtered features. Figure S3: ROC Curve Analysis of Key Metabolite Biomarkers Distinguishing HN and LW Pigs. Receiver operating characteristic (ROC) curves for three representative differential metabolites: 4-ethyl-2-heptylthiazole, methylisatoid, and picolinic acid. All three metabolites achieved an AUC (area under the curve) value of 1.000, indicating perfect discrimination between Huainan and Large White pigs based on their intestinal metabolite profiles; Table S1: Summary of 16S rRNA Sequencing Data and OTU Classification Results; Table S2: Differential Metabolites Identified Between HN and LW Pigs in Rectal and Colonic Contents.

## Abbreviations

The following abbreviations are used in this manuscript:

ABC transporter	ATP-Binding Cassette Transporter
AUC	Area Under the Curve
HN	Huainan pig
HMDB	Human Metabolome Database
IMF	Intramuscular Fat
KEGG	Kyoto Encyclopedia of Genes and Genomes
LDA	Linear Discriminant Analysis
LEfSe	Linear Discriminant Analysis Effect Size
LPS	Lipopolysaccharide
LW	Large White pig
OPLS-DA	Orthogonal Partial Least Squares Discriminant Analysis
OTU	Operational Taxonomic Unit
PCoA	Principal Coordinates Analysis
PICRUSt2	Phylogenetic Investigation of Communities by Reconstruction of Unobserved States 2
PPARγ	Peroxisome Proliferator-Activated Receptor Gamma
ROC	Receiver Operating Characteristic
SCFA	Short-Chain Fatty Acid
VIP	Variable Importance in Projection

## References

[1] McCormack U.M., Curião T., Buzoianu S.G., Prieto M.L., Ryan T., Varley P., Crispie F., Magowan E., Metzler-Zebeli B.U., Berry D. (2017). Exploring a Possible Link Between the Intestinal Microbiota and Feed Efficiency in Pigs. Appl. Environ. Microbiol..

[2] Mach N., Berri M., Estellé J., Levenez F., Lemonnier G., Denis C., Leplat J.J., Chevaleyre C., Billon Y., Doré J. (2015). Early-life establishment of the swine gut microbiome and impact on host phenotypes. Environ. Microbiol. Rep..

[3] Lee J.H., Kim S., Kim E.S., Keum G.B., Doo H., Kwak J., Pandey S., Cho J.H., Ryu S., Song M. (2023). Comparative analysis of the pig gut microbiome associated with the pig growth performance. J. Anim. Sci. Technol..

[4] Zhang Q., Du M., Wei S., Zhu L., Yan R., Jin M., Wang Y. (2025). Variation of meat quality and relationship to gut microbiota among different pig breeds. Microb. Biotechnol..

[5] Wang Y., Wang M., Chen J., Li Y., Kuang Z., Dende C., Raj P., Quinn G., Hu Z., Srinivasan T. (2023). The gut microbiota reprograms intestinal lipid metabolism through long noncoding RNA Snhg9. Science.

[6] Gardiner G.E., Metzler-Zebeli B.U., Lawlor P.G. (2020). Impact of intestinal microbiota on growth and feed efficiency in pigs: A review. Microorganisms.

[7] Ma J., Duan Y., Li R., Liang X., Li T., Huang X., Yin Y., Yin J. (2022). Gut microbial profiles and the role in lipid metabolism in Shaziling pigs. Anim. Nutr..

[8] Bergamaschi M., Tiezzi F., Howard J., Huang Y.J., Gray K.A., Schillebeeckx C., McNulty N.P., Maltecca C. (2020). Gut microbiome composition differences among breeds impact feed efficiency in swine. Microbiome.

[9] Chen H., Nwe P.K., Yang Y., Rosen C.E., Bielecka A.A., Kuchroo M., Cline G.W., Kruse A.C., Ring A.M., Crawford J.M. (2019). A forward chemical genetic screen reveals gut microbiota metabolites that modulate host physiology. Cell.

[10] Wang M., Zhang L., Liu Z., Guo A., Yang G., Yu T. (2025). Host-microbiota interactions in the pathogenesis of porcine fetal mummification. Microorganisms.

[11] Colosimo D.A., Kohn J.A., Luo P.M., Piscotta F.J., Han S.M., Pickard A.J., Rao A., Cross J.R., Cohen L.J., Brady S.F. (2019). Mapping interactions of microbial metabolites with human G-protein-coupled receptors. Cell Host Microbe.

[12] Jian Z., Zeng L., Xu T., Sun S., Yan S., Zhao S., Su Z., Ge C., Zhang Y., Jia J. (2022). The intestinal microbiome associated with lipid metabolism and obesity in humans and animals. J. Appl. Microbiol..

[13] Oh H.Y.P., Visvalingam V., Wahli W. (2019). The PPAR-microbiota-metabolic organ trilogy to fine-tune physiology. FASEB J..

[14] Chen H., Wang S.H., Li H.L., Zhou X.B., Zhou L.W., Chen C., Mansell T., Novakovic B., Saffery R., Baker P.N. (2024). The attenuation of gut microbiota-derived short-chain fatty acids elevates lipid transportation through suppression of the intestinal HDAC3-H3K27ac-PPAR-γ axis in gestational diabetes mellitus. J. Nutr. Biochem..

[15] Funabashi M., Grove T.L., Wang M., Varma Y., McFadden M.E., Brown L.C., Guo C., Higginbottom S., Almo S.C., Fischbach M.A. (2020). A metabolic pathway for bile acid dehydroxylation by the gut microbiome. Nature.

[16] Du J., Gan M., Xie Z., Du G., Luo Y., Liu B., Zhu K., Chen L., Zhao Y., Niu L. (2023). A comparative study on the growth performance and gut microbial composition of Duroc and Yorkshire boars. Genes.

[17] Li J., Li Y., Cheng M., Ye F., Li W., Wang C., Huang Y., Wu Y., Xuan R., Liu G. (2022). Gut microbial diversity among Yorkshire, Landrace and Duroc boars and its impact on semen quality. AMB Express.

[18] Zhang S., Zhang H., Zhang C., Wang G., Shi C., Li Z., Gao F., Cui Y., Li M., Yang G. (2024). Composition and evolutionary characterization of the gut microbiota in pigs. Int. Microbiol..

[19] Wang W., Wang D., Zhang X., Liu X., Niu X., Li S., Huang S., Ran X., Wang J. (2024). Comparative transcriptome analysis of longissimus dorsi muscle reveal potential genes affecting meat trait in Chinese indigenous Xiang pig. Sci. Rep..

[20] Murga-Garrido S.M., Hong Q., Cross T.L., Hutchison E.R., Han J., Thomas S.P., Vivas E.I., Denu J., Ceschin D.G., Tang Z.Z. (2021). Gut microbiome variation modulates the effects of dietary fiber on host metabolism. Microbiome.

[21] Shen J., Zhang J., Zhao Y., Lin Z., Ji L., Ma X. (2022). Tibetan pig-derived probiotic Lactobacillus amylovorus SLZX20-1 improved intestinal function via producing enzymes and regulating intestinal microflora. Front. Nutr..

[22] (2023). Welfare Criteria for Animals to Be Slaughtered.

[23] Rognes T., Flouri T., Nichols B., Quince C., Mahé F. (2016). VSEARCH: A versatile open source tool for metagenomics. PeerJ.

[24] Edgar R.C., Haas B.J., Clemente J.C., Quince C., Knight R. (2011). UCHIME improves sensitivity and speed of chimera detection. Bioinformatics.

[25] Wright R.J., Langille M.G.I. (2025). PICRUSt2-SC: An update to the reference database used for functional prediction within PICRUSt2. Bioinformatics.

[26] Langille M.G., Zaneveld J., Caporaso J.G., McDonald D., Knights D., Reyes J.A., Clemente J.C., Burkepile D.E., Vega Thurber R.L., Knight R. (2013). Predictive functional profiling of microbial communities using 16S rRNA marker gene sequences. Nat. Biotechnol..

[27] Shokryazdan P., Sieo C.C., Kalavathy R., Liang J.B., Alitheen N.B., Faseleh Jahromi M., Ho Y.W. (2014). Probiotic potential of Lactobacillus strains with antimicrobial activity against some human pathogenic strains. Biomed Res. Int..

[28] Phua W.W.T., Wong M.X.Y., Liao Z., Tan N.S. (2018). An aPPARent functional consequence in skeletal muscle physiology via peroxisome proliferator-activated receptors. Int. J. Mol. Sci..

[29] Kataoka K. (2016). The intestinal microbiota and its role in human health and disease. J. Med. Invest..

[30] Egamberdiyeva D. (2005). Characterization of Pseudomonas species isolated from the rhizosphere of plants grown in serozem soil, semi arid region of Uzbekistan. Sci. World J..

[31] Pu G., Hou L., Du T., Zhou W., Liu C., Niu P., Wu C., Bao W., Huang R., Li P. (2023). Increased proportion of fiber-degrading microbes and enhanced cecum development jointly promote host to digest appropriate high-fiber diets. mSystems.

[32] Lacourt T.E., Vichaya E.G., Chiu G.S., Dantzer R., Heijnen C.J. (2018). The high costs of low-grade inflammation: Persistent fatigue as a consequence of reduced cellular-energy availability and non-adaptive energy expenditure. Front. Behav. Neurosci..

[33] Rodríguez-Cano M.M., González-Gómez M.J., Sánchez-Solana B., Monsalve E.M., Díaz-Guerra M.M., Laborda J., Nueda M.L., Baladrón V. (2020). NOTCH receptors and DLK proteins enhance brown adipogenesis in mesenchymal C3H10T1/2 cells. Cells.

[34] Uno Y., Morikuni S., Shiraishi M., Asano A., Kawaguchi H., Murayama N., Yamazaki H. (2022). A comprehensive analysis of six forms of cytochrome P450 2C (CYP2C) in pigs. Xenobiotica.

[35] Chiang J.Y.L., Ferrell J.M. (2020). Up to date on cholesterol 7 alpha-hydroxylase (CYP7A1) in bile acid synthesis. Liver Res..

[36] Wang Z., Zhao Y. (2018). Gut microbiota-derived metabolites in cardiovascular health and disease. Protein Cell.

[37] Lyu M., Bai Y., Orihara K., Miyanaga K., Yamamoto N. (2023). GAPDH released from Lactobacillus johnsonii MG enhances barrier function by upregulating genes associated with tight junctions. Microorganisms.

[38] Jin S.J., Song Y., Park H.S., Park K.W., Lee S., Kang H. (2022). Harmine inhibits multiple TLR-induced inflammatory expression through modulation of NF-κB p65, JNK, and STAT1. Life.

