# Supplementary figures and images for: Impact of the Gut Microbiota–Metabolite Axis on Intestinal Fatty Acid Absorption in Huainan Pigs
Source: Microorganisms. 2025 Jul 8;13(7):1609. doi: 10.3390/microorganisms13071609 (PMC12299282; doi:10.3390/microorganisms13071609)

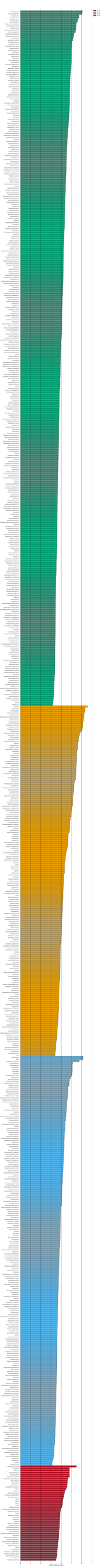

Supplement: Supplementary file 1 [file microorganisms-13-01609-s001.zip › supplementary_figure_s1 lefse_LDA.pdf]

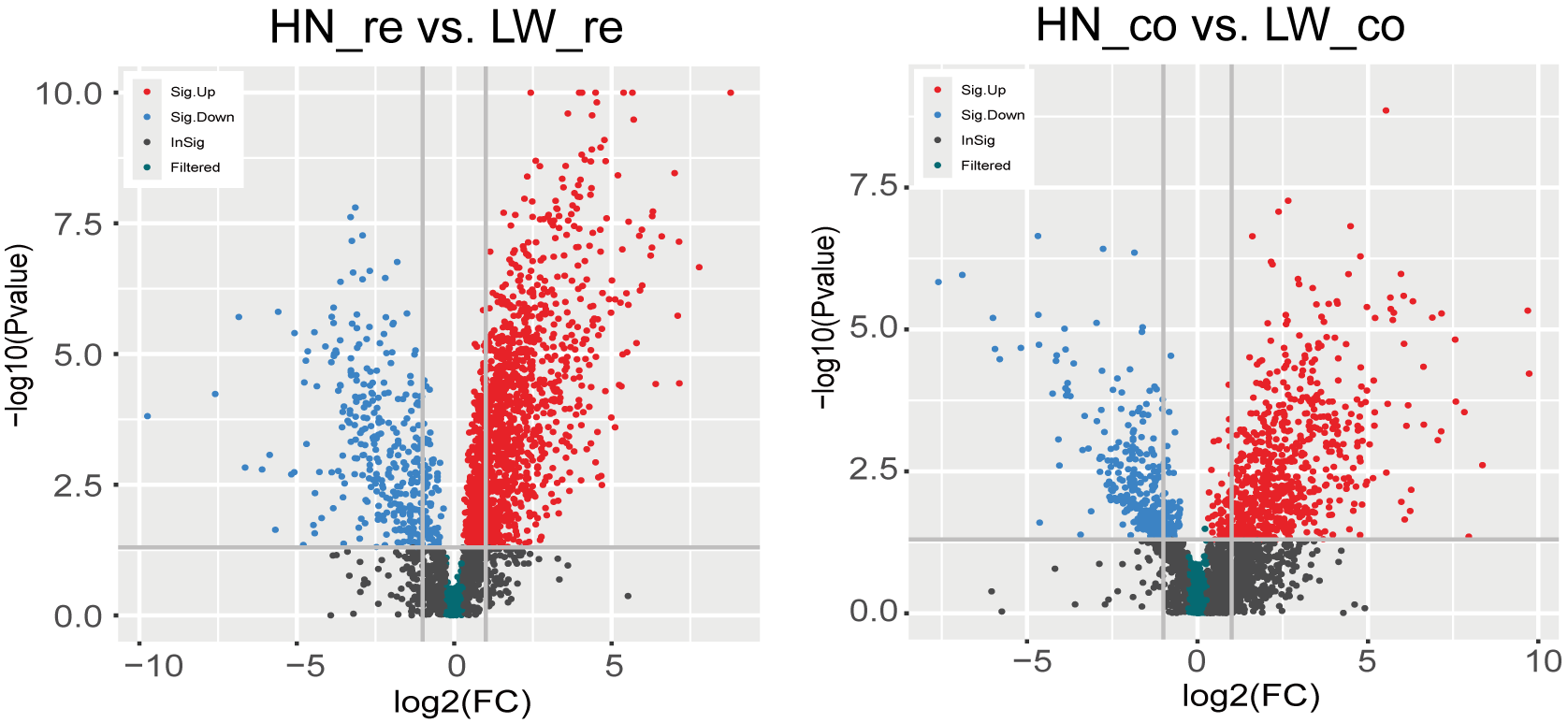

Supplement: Supplementary file 1 [file microorganisms-13-01609-s001.zip › supplementary_figure_s2.tif]

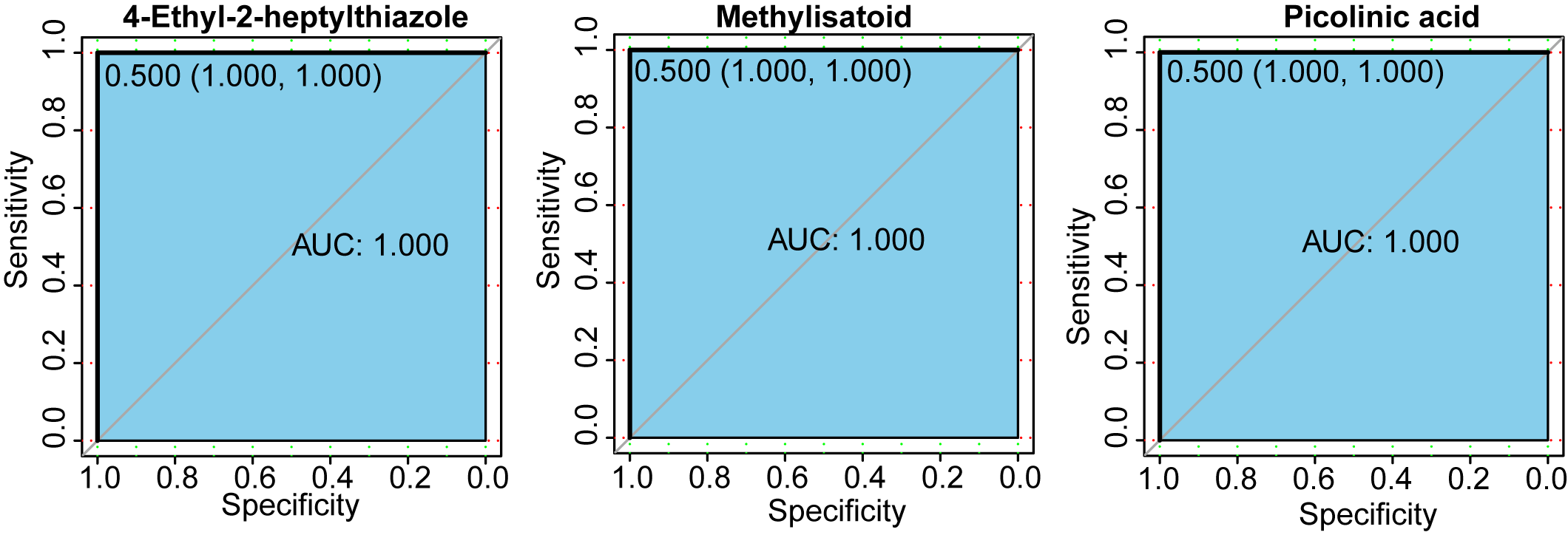

Supplement: Supplementary file 1 [file microorganisms-13-01609-s001.zip › supplementary_figure_s3.tif]
